# Reveal the Patterns of Prescriptions for Recurrent Respiratory Tract Infections' Treatment Based on Multiple Illustrious Senior Traditional Chinese Medicine Practitioners

**DOI:** 10.1155/2023/7982927

**Published:** 2023-05-25

**Authors:** Bochuan Wang, Jiang Zhou, Bing He, Huiyang Shi, Xue Liang, Zhiqiang Zhang, Changyong Luo, Chen Bai, Yixuan Ao, He Yu, Xiaohong Gu

**Affiliations:** ^1^School of Traditional Chinese Medicine, Beijing University of Chinese Medicine, Beijing, China; ^2^Hospital of Chengdu University of Traditional Chinese Medicine, Chengdu, Sichuan, China; ^3^Dongzhimeng Hospital Beijing University of Chinese Medicine, Beijing University of Chinese Medicine, Beijing, China; ^4^The Second Hospital of Liaoning University of Traditional Chinese Medicine, Shenyang, Liaoning, China; ^5^Jilin Academy of Traditional Chinese Medicine, Changchun, Jilin, China; ^6^Beijing Tcmages Pharmaceutical Co., Ltd., Beijing, China; ^7^Dongfang Hospital Beijing University of Chinese Medicine, Beijing University of Chinese Medicine, Beijing, China

## Abstract

**Background:**

Recurrent respiratory tract infections (RRTIs) are one of the most common diseases in children and adolescents. The causes of RRTIs are various. In addition to the factors related to infection, basic diseases such as respiratory system, immune system, and digestive system are also involved. The cost of patients' frequent medical treatment and hospitalization has been deemed to be a heavy burden to the society and family. In China, traditional Chinese medicine (TCM) is commonly used to treat RRTIs. TCM treatment has been appraised to be effective, for reducing the number of hospital stays. Illustrious senior TCM practitioners of pediatrics are recognized as a group of outstanding physicians with significantly better patient outcomes. However, different illustrious senior TCM practitioners can lead to differences in treatment strategies due to factors such as region, prescription theory, and individual differences of patients. This makes it difficult for the experience of illustrious senior TCM practitioners to be popularized. However, there have been no prescription mining studies for the treatment of RRTIs based on different and multiple illustrious senior TCM practitioners. We explored the core prescriptions and drug mechanisms through data mining based on the prescriptions of illustrious senior TCM practitioners treating RRTIs from different clinical settings. This is important to promote the effective treatment of RRTIs with TCM. The objective of this study is to reveal the strategies (core prescriptions) from the prescriptions of multiple illustrious senior TCM practitioners for the treatment of RRTIs. We hope that this core prescription can help all TCM pediatricians to improve RRTIs children's outcome. Meanwhile, it could provide a new way for researchers to study the treatment of RRTIs.

**Methods:**

In this study, we prospectively collected 400 children's prescriptions with RRTIs receiving TCM treatment from four illustrious senior TCM practitioners in different hospitals. We described and analyzed the characteristics of TCM prescriptions. The prescription regularity was analyzed by hierarchical clustering and association rules. Network pharmacology methods has been used to reveal the pathway mechanism of core prescriptions which have been mined and visualized with the help of SymMap, Genecards, KEGG, Metascape databases, and *R*. The execution of all methods was completed in May 2022.

**Results:**

According to RRTIs multiple clinical syndromes, five new prescriptions were obtained based on illustrious senior TCM practitioners. Among them, the prescription composed of *Scutellariae radix* (Huangqin), *Armeniacae semen amarum* (Kuxingren), *Peucedani radix* (Qianhu), and *Pheretima* (Dilong) is the core strategy for the treatment of RRTIs. Cold herbs and heat herbs in the core prescription are approximately equal. *Scutellariae radix* (Huangqin) was dominant, and other herbs exert synergistic effects. The core prescription covered 76 pathways and 226 herb-disease genes. It promotes the differentiation of Th1, Th2, and Th17 cells and the secretion of inflammatory factors through toll-like receptor signaling pathway in the immune system, T cell receptor signaling pathway, and PPAR signaling pathway in the endocrine system, thereby exerting immune regulation and anti-inflammation.

**Conclusion:**

In this study, we revealed the prescription regularity of TCM in the treatment of RRTIs and analyzed the mechanism of core prescriptions, which provided new ideas for the treatment of RRTIs.

## 1. Introduction

Recurrent respiratory infections (RRTIs) refer to the upper and lower respiratory tract infections that occur frequently and exceed the normal range within one year [1]. RRTIs are one of the most common diseases in children and adolescents [2]. The increased incidence of RRTIs in children is mainly related to age, immune system conditions, concomitant diseases and allergies, and overuse of antibiotics [3]. Children with RRTIs have difficulty removing pathogens from the body, which may contribute to the recurrence of pulmonary infections. RRTIs can seriously impact the wellbeing and health of children, moreover, some of the RRTI patients may suffer repeated wheezing, malnutrition, anemia, growth retardation, and even decreased pulmonary function [4]. RRTIs in childhood may increase susceptibility to diseases in adulthood, such as asthma, diabetes, chronic bronchitis, and emphysema [5]. Data from the World Health Organization show that RRTIs occur primarily in children under 5 years of age and account for 10%∼30% of all pediatric respiratory infections. The incidence of RRTIs is rising every year. Children with RRTIs immunodeficiency could take a few treatments such as antibody replacement therapy, however, always with huge cost [6]. This puts a heavy burden on the patient's family and society. Therefore, it is a pressing issue to develop an effective and affordable drug for RRTIs.

As an ancient medicine, traditional Chinese medicine (TCM) has accumulated precious experience for clinical treatment and medical research in various fields for a long time [7]. TCM has played an important role as a complementary alternative therapy in East Asia. Researchers in many countries around the world have paid attention to the potential value of TCM in treating chronic diseases. Studies have shown that TCM can reduce the incidence of RRTIs, significantly improve the clinical symptoms of RRTIs, and reduce the number of recurrent respiratory tract infections, while having good safety [8, 9]. In clinical, TCM physicians, especially illustrious senior TCM practitioners, usually choose more precise and individualized strategies. Therefore, the efficacy of illustrious senior TCM practitioners in the treatment of RRTIs is more prominent, while it has been the focus of attention of pediatricians and researchers. But to some extent, this will also bring difficulties to the promotion of clinical strategies. At present, the research on the basic therapeutic principle, prescription compatibility, and prescription mechanism of illustrious senior TCM practitioners has attracted much attention [10]. Therefore, the study of the prescription mechanism of illustrious senior TCM practitioners in the treatment of RRTIs will help to improve the efficacy of RRTIs as well as the popularization of illustrious senior TCM practitioners' experience.

Data mining has been widely used in disease diagnosis, syndrome differentiation, and prescription analysis [11]. It is an efficient idea to analyze the prescription regularity of TCM from large-scale TCM medical records and discover the potential relationship between herbs and diseases. In recent years, system pharmacology has been proved to be a useful tool for further exploring the pharmacological mechanisms in TCM [12]. It integrates phytochemistry, pharmacology, and bioinformatics and effectively connects the bridge between modern medicine and traditional medicine. By analyzing the herb-disease targets and enrichment pathways of the prescriptions, we can understand the mechanism of the core prescription (CP) more clearly.

**Figure 1:**
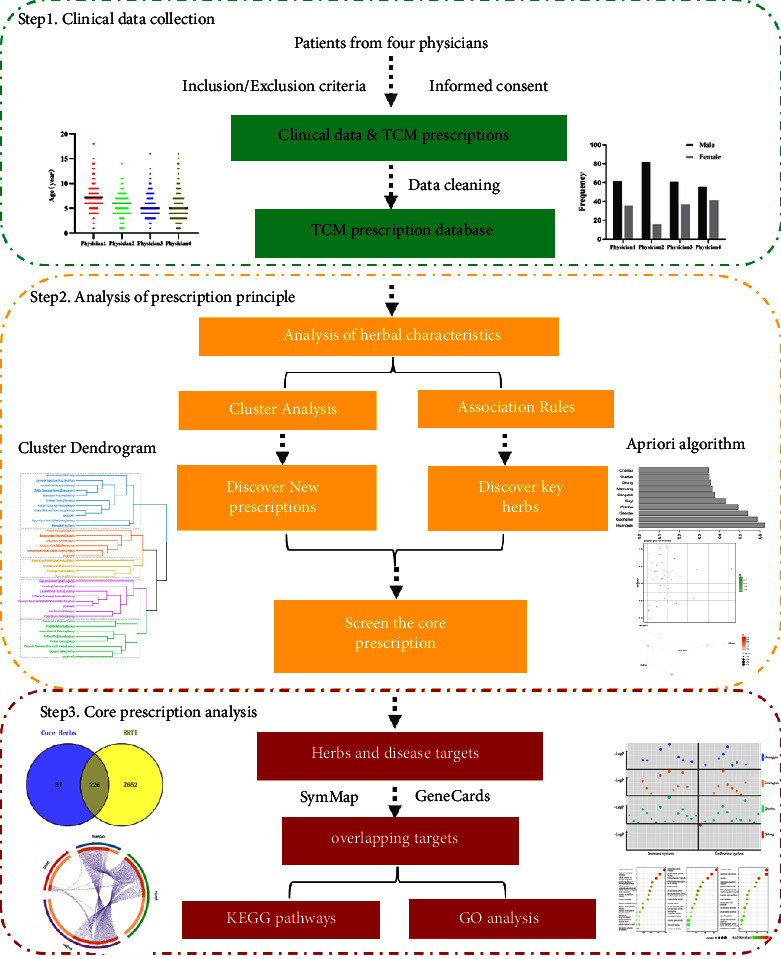
Workflow of the approach.

Step 1 presents the source and the basic characteristics of the data. Step 2 shows how to use data mining (clustering analysis and association rules) to get the core prescriptions. Step 3 introduces the mechanism of core prescriptions by using network pharmacology (multisource database). The workflow chart is shown in Figure 1.

## 2. Related Work

Previous studies mainly focused on the case report of one doctor in a single hospital center. In this study, we prospectively collected clinical cases of RRTIs in children in multiple hospitals based on real-world records. The four pediatric illustrious senior TCM practitioners who participated in the study were renowned nationwide. They come from three regions of China (northeast, north, and southwest) and four provinces (Jilin, Liaoning, Beijing, and Sichuan). They have been engaged in the treatment of pediatric diseases for more than forty years. The patients can achieve a better outcome from their treatment, and they have high reputations in this field [13, 14]. We have fully read and analyzed the published case reports and clinical studies of these pediatric illustrious senior TCM practitioners in the previous period, so as to ensure that their clinical strategies will not be misunderstood or missed. We explored core prescriptions by analyzing prescriptions of RRTIs through descriptive statistics, hierarchical clustering, and association rules. We exchanged and discussed the core prescriptions obtained by data mining with pediatric illustrious senior TCM practitioners. The core prescription has been approved by them, which is in line with their treatment strategies in the real-world. Subsequently, targets for core prescriptions were screened from the database. Metascape was used to analyze the KEGG pathway and GO process, thereby increasing the understanding of the pathogenesis and pathology of RRTIs [15].  Table S1 shows the difference between related work and previous studies. To some extent, this study can promote the medical community's understanding and acceptance of the TCM therapy.

## 3. Materials and Methods

### 3.1. Clinical Data Collection

In four clinical centers (Dongzhimen Hospital of Beijing University of Traditional Chinese Medicine, Second Affiliated Hospital of Liaoning University of Traditional Chinese Medicine, Affiliated Hospital of Chengdu University of Traditional Chinese Medicine, and First Clinical Hospital of Jilin Academy of Traditional Chinese Medicine), we prospectively collected the prescriptions of four pediatric illustrious senior TCM practitioners for RRTIs from November 2019 to October 2021, with 100 cases in each center.

The respiratory group of the Pediatric Society of Chinese Medical Association formulated the diagnostic criteria of RRTIs (Table 1).

#### 3.1.1. Inclusion Criteria

The inclusion criteria include the following: (1) children meeting the above diagnostic criteria for RRTIs; (2) the age of children is between 1 and 17 years; (3) the children or the guardian of the children sign the informed consent form.

#### 3.1.2. Exclusion Criteria

The exclusion criteria include the following: (1) children suffer from severe primary disease; (2) children are participating in other clinical trials.

We collected the general clinical data of these cases, including the patient's gender, age, diagnosis, and TCM prescriptions. All data were entered into Microsoft Excel 2016 to establish a database for the treatment of RRTIs by four physicians. The prescription herbs were subsequently further standardized in the following manner. In accordance with the Chinese Pharmacopoeia of the People's Republic of China (2020 edition) and “Chinese Materia Medica,” the properties, flavours, and meridian tropism of the medicinal herbs in these prescriptions were further supplemented. Three researchers were assigned to complete the information entry work separately. The research protocol was reviewed and approved by the Ethics Committee of Beijing University of Chinese Medicine (ethics batch no. 2019BZHYLL0204). The researchers followed the protocol strictly.

### 3.2. General Clinical Information and Prescription Herbs Analyzing

We used Microsoft Office Excel 2016 for statistical analysis of the patient's age and gender distribution. GraphPad Prism9 and Adobe Illustrator are used for graphics. Firstly, we analyzed the property, flavours, and meridian tropism of the prescription herbs, and then used hierarchical clustering to find new prescriptions. Finally, we used association rules to screen out the core herbs. RStudio 1.4.1717 due to the visualization of the above method.

#### 3.2.1. Descriptive Analysis: Herbal Properties, Flavours, and Meridian Tropism

Descriptive analysis of herbs' characteristics, including three parts: properties, flavours, and meridian tropism. The properties consist of cold, cool, mild, warm, and hot. The flavours consist of sour, astringent, salty, sweet, bitter, pungent, and light. There are twelve items of meridians: spleen, liver, heart, kidney, pericardium, stomach, urinary bladder, large intestine, small intestine, trijiao, gallbladder, and lung. The top 10 high-frequency herbs of each physician were selected for visual analysis of herbal properties and flavours.

#### 3.2.2. Cluster Analysis: Discovering New Prescriptions

The hierarchical clustering algorithm was used to cluster each herb. According to the similarity measurement among the objects, stable and regular new categories could be obtained in many individualized prescriptions. In order to reduce the possible bias of the difference of each physician on the core prescription, we selected the top 10 herbs in each physician's prescription and deleted the duplicate values, finally obtaining 33 Chinese herbs. The *x* and *y* in the following algorithm formula represent different herbs, respectively. The similarity between herbs was calculated by using the Euclidean distance. Draw the tree diagram, different colors labeled with different categories.

(1) $$\begin{array}{c} d\left(x,y\right)=\sqrt{\overset{n}{\underset{k=1}{\sum }}{\left(x_{k}-y_{k}\right)}^{2}}\text{.} \end{array}$$

#### 3.2.3. Association Rules: Screening the Core Prescription

Apriori algorithm is an association rule algorithm for frequent itemsets. We used the apriori algorithm to analyze the prescriptions and found the core herbs. Each prescription is equivalent to a transaction, and each herb is equivalent to an itemset. Through calculating frequent itemsets, we screened the association rules between herbs and finally obtained the core herbs. Core prescriptions were obtained by filtering the rules with top “Support.” *X* and *Y* in the following formula represent the frequent itemsets of one or more herbs. “Support” refers to the proportion of transactions containing *X* and *Y* (*P* (*X*∪*Y*)) in all transactions (*P* (*I*)), that is, the probability of simultaneous occurrence of *X* and *Y*. “Confidence” refers to proportion of transactions with *X* that contain both *Y* transactions, that is, the probability of *Y* occurring at the same time when *X*. “Lift” refers to the ratio of “proportion of transactions with *X* that include *Y* at the same time” to “proportion of transactions with *Y*.” “Lift” reflects the correlation between *X* and *Y*. “Lift” greater than 1 and higher indicates a higher positive correlation; “Lift” less than 1 and lower indicates a higher negative correlation, and “Lift” equal to 1 indicates no correlation.

(2) $$\begin{array}{c} Support\left(X=>Y\right)=\frac{P\left(X\cup Y\right)}{P\left(I\right)}\text{,}\\ Confidence\left(X=>Y\right)=\frac{P\left(X\cup Y\right)}{P\left(X\right)}\text{,}\\ Lift\left(X=>Y\right)=\frac{P\left(X\cup Y\right)}{P\left(X\right)P\left(Y\right)}\text{.} \end{array}$$

### 3.3. Analysis of Prescription Mechanisms

#### 3.3.1. Chemical Composition and Targets of the Core Prescription (CP)

In this study, we used the SymMap database (http://www.symmap.org/), to acquire the molecular targets of each herb in the core prescriptions.

#### 3.3.2. Disease Targets Database Building

In this study, we used the GeneCards database (http://www.genecards.org/), to acquire relevant disease targets for RRTIs.

### 3.4. Functional Annotation and Enrichment Analysis

Metascape (http://metascape.org/gp/index.html#/main/step1) was used to analyze the targets obtained, the species for selection was “Homo sapiens,” and custom analysis mode was used, set *P* value < 0.01 and min overlap > 3, the KEGG pathway result was obtained. Then, only items that also have −log (*P* value) > 5 and belong to more than 5% of the target of the category were kept. RStudio's ggplot2 was used to draw the remaining items into a bubble chart. Finally, the bubble graphs of each herb was combined.

## 4. Results

### 4.1. Patient Characteristics

**Figure 2:**
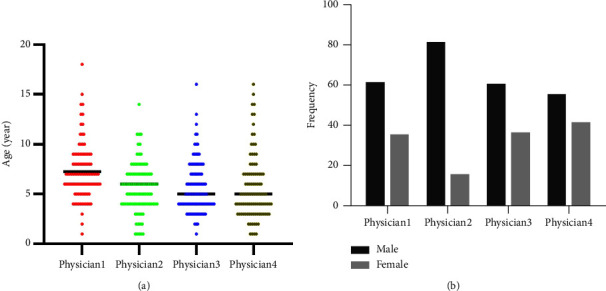
General information of patients. (a) Gender distribution of patients. (b) Age distribution of patients.

In this study, 400 qualified patients with complete information of their age, gender, and prescription information were selected for a total of 400 consultations, with an average of 1 consultations per patient. The male to female ratios for the four physicians were 1.70 : 1/4.88 : 1/1.63 : 1 and 1.33 : 1. The average age of the patients is 7.3, 5.6, 5.7, and 5.6 years, respectively, Figure 2.

### 4.2. Analysis of Herbal Characteristics

**Figure 3:**
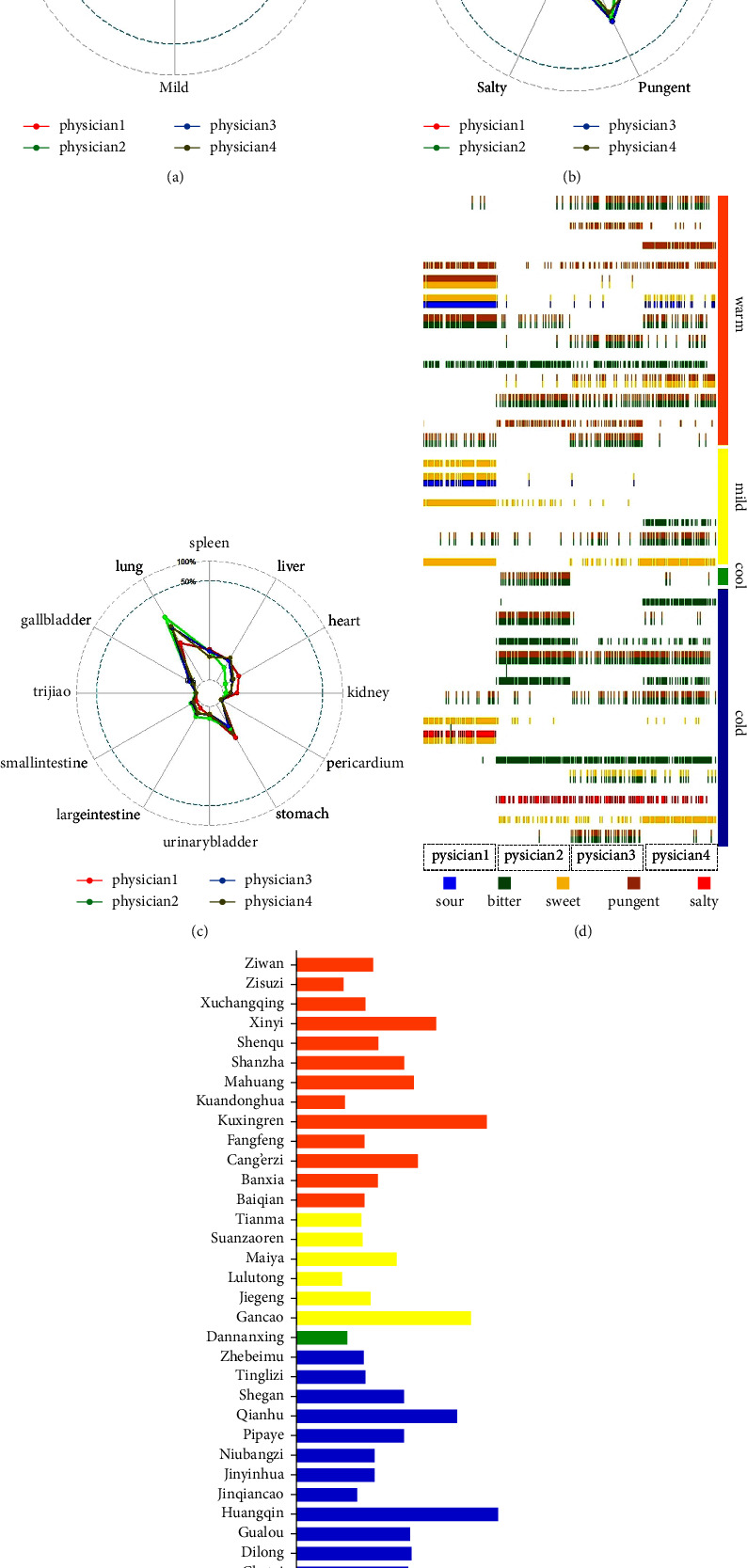
(a) Percentage of herbal properties; (b) percentage of herbal flavours; (c) percentage of meridian; (d) the *X*-axis was the medical record number of the four physicians' patients, and the *Y*-axis was herbs. The characteristics of herbs consisted of cold, cold, mild, and warm, and the color of the line in the coordinates, respectively, corresponded to the five flavours of sour, bitter, sweet, pungent, and salty of the herbs. Figure (e) is the detailed name and frequency corresponding to the herbs in Figure (d).

Among the 400 prescriptions, there are 186 kinds of herbs. The frequency of herbs is 6324 times in total. The radar chart of Figure 3(a) shows that the frequency of cold and warm herbs is the highest, which is 2870 and 2164 times. The frequency of mild and cool herbs is 1096 and 194, respectively. All prescriptions do not contain hot medicine. Physician 1 prefers mild herbs compared to other physicians, and Physician 2 prefers cold herbs. The radar chart of Figure 3(b) shows that the most frequent flavours of herbs are bitter, sweet, and pungent, which are 3206, 2694, and 2647 times, respectively, and less than 400 times were recorded for the rest of the flavours. Physician 1 prefers sweet herbs compared to other physicians, and Physician 2 prefers bitter herbs. The radar chart of Figure 3(c) shows that the most frequent meridians of herbs are the lung meridian, stomach meridian, spleen meridian, liver meridian, and heart meridian, which are 4496, 2355, 2029, 1786, and 1005 times, respectively. Figures 3(d) and 3(e) show herbal properties, flavours characteristics of the top 10 frequency herbs in each physician's prescriptions.  Table 2 shows the frequency of herbal characteristics in four physicians' prescriptions.

### 4.3. Hierarchical Cluster Analysis

We analyzed the top 10 herbs in the prescription of each physician, including 33 herbs in total. Herbal frequency, properties and flavours characteristics, and affiliated physicians of the herbs are shown in Table 3. Among them, the frequency of *Scutellariae radix* (Huangqin) was 248 times at most, and the frequency of *Armeniacae semen amarum* (Kuxingren) was 234 times.

Among them, *Scutellariae radix* (Huangqin) appeared 248 times most frequently, and *Armeniacae semen amarum* (Kuxingren) appeared 234 times. The frequency of other herbs in descending order were *Glycyrrhizae radix et rhizoma* (Gancao), *Peucedani radix* (Qianhu), *Magnoliae flos* (Xinyi), *Xanthii fructus* (Cang'erzi), *Ephedrae herba* (Mahuang), and *Pheretima* (Dilong). These herbs are commonly used to treat RRTIs.

**Figure 4:**
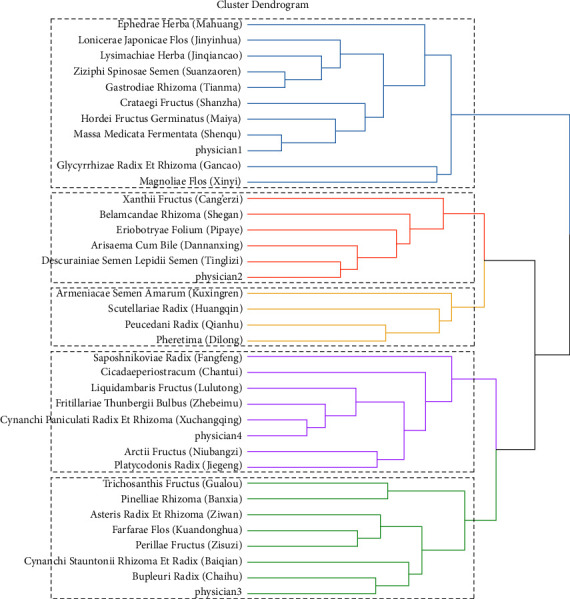
Herbs hierarchical clustering results: 33 herbs were divided into five new prescriptions, and each prescription was displayed in a different color.

We divided these herbs into five new prescriptions according to the TCM theory. There were four prescriptions corresponding to different physicians, respectively (Figure 4). Prescription 1 was assigned to Physician 1 and included *Ephedrae herba* (Mahuang), *Lonicerae japonicae flos* (Jinyinhua), *Lysimachiae herba* (Jinqiancao), *Ziziphi spinosae semen* (Suanzaoren), *Gastrodiae rhizoma* (Tianma), *Crataegi fructus* (Shanzha), *Hordei fructus germinatus* (Maiya), *Massa medicata fermentata* (Shenqu), *Glycyrrhizae radix et rhizoma* (Gancao), and *Magnoliae flos* (Xinyi). Prescription 2 was assigned to Physician2 and included *Xanthii fructus* (Cang'erzi), *Belamcandae rhizoma* (Shegan), *Eriobotryae folium* (Pipaye), *Arisaema cum bile* (Dannanxing), and *Descurainiae semen lepidii semen* (Tinglizi). Prescription 3 was divided into a separate category, without any particular preference for any physicians, and included *Scutellariae radix* (Huangqin), *Armeniacae semen amarum* (Kuxingren), *Peucedani radix* (Qianhu), and *Pheretima* (Dilong). Prescription 4 was assigned to Physician 4 and included *Saposhnikoviae radix* (Fangfeng), *Cicadaeperiostracum* (Chantui), *Liquidambaris fructus* (Lulutong), *Fritillariae thunbergii bulbus* (Zhebeimu), *Cynanchi paniculati radix et rhizoma* (Xuchangqing), *Arctii fructus* (Niubangzi), and *Platycodonis radix* (Jiegeng). Prescription 5 was assigned to Physician 3 and included *Trichosanthis fructus* (Gualou), *Pinelliae rhizoma* (Banxia), *Asteris radix et rhizoma* (Ziwan), *Farfarae flos* (Kuandonghua), *Perillae fructus* (Zisuzi), *Cynanchi stauntonii rhizoma et radix* (Baiqian), and *Bupleuri radix* (Chaihu).

### 4.4. Association Rule Analysis

**Figure 5:**
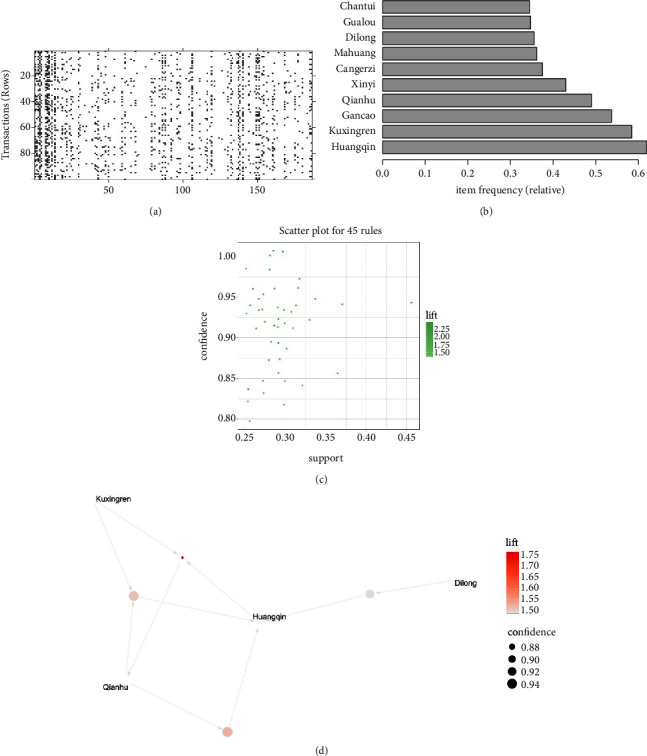
Herbs association rule results: (a) the chart of frequent items. (b) The chart of top 10 items. (c) The chart of top 45 rules. (d) The chart of association rules in the top 4 support levels.

First, we transform the prescriptions data into frequent itemsets, and the top 10 frequent items were *Scutellariae radix* (Huangqin), *Armeniacae semen amarum* (Kuxingren), *Glycyrrhizae radix et rhizoma* (Gancao), *Peucedani radix* (Qianhu), *Magnoliae flos* (Xinyi), *Xanthii fructus* (Cang'erzi), *Ephedrae herba* (Mahuang), *Pheretima* (Dilong), *Trichosanthis fructus* (Gualou), and *Cicadaeperiostracum* (Chantui) (Figure 5(b)). Set support = 0.25, confidence = 0.8, min len = 2, and max len = 5. There were 45 rules (Figure 5(c)), see Table S2 for specific association rules. The top 4 rules of support were selected for visual presentation. From Figure 5(d), it can be seen that *Scutellariae radix* (Huangqin), *Armeniacae semen amarum* (Kuxingren), *Peucedani radix* (Qianhu), and *Pheretima* (Dilong) were the herbs with the strongest association and were in the core position. At the same time, we found that the core herbs were highly overlapped with New Prescription 3, and could become the core prescription. We could also refer to those herbs as the core prescription (CP) for the treatment of RRTIs.

### 4.5. Overall Targets of the CP for RRTIs Treatment

**Figure 6:**
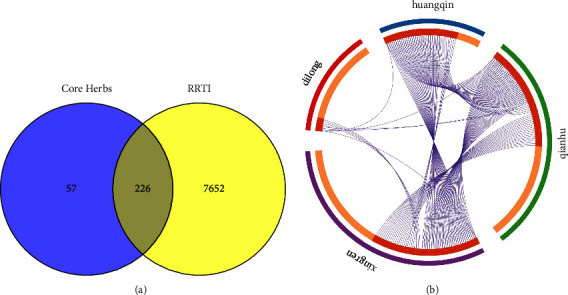
(a) Venn diagram of RRTIs' targets and core herbs' targets. (b) TCM targets show, the connection between different TCM targets, the blue color of the outer circle represents *Scutellariae radix* (Huangqin), the green color represents *Peucedani radix* (Qianhu), the purple color represents *Armeniacae semen amarum* (Kuxingren), and the red color represents *Pheretima* (Dilong). The inner circle represents the list of genes. The purple curve connects the same genes. Identical genes of multiple herbs are shown in dark orange. Genes that appeared only once are shown in light orange.

We obtained 283 targets (*P* < 0.001) of 4 herbs in CP from the SymMap database. We obtained a total of 7878 targets related to RRTIs from GeneCards database. We intersected the targets of the herbs with the targets of RRTIs to obtain a total of 226 interactive targets, including 51 targets of *Scutellariae radix* (Huangqin), 113 targets of *Armeniacae semen amarum* (Kuxingren), 106 targets of *Peucedani radix* (Qianhu), and 51 targets of *Pheretima* (Dilong) (Figure 6), see Table S3 for specific disease-herb interaction targets.

### 4.6. Functional and Enrichment Analysis of Core Herbs

**Figure 7:**
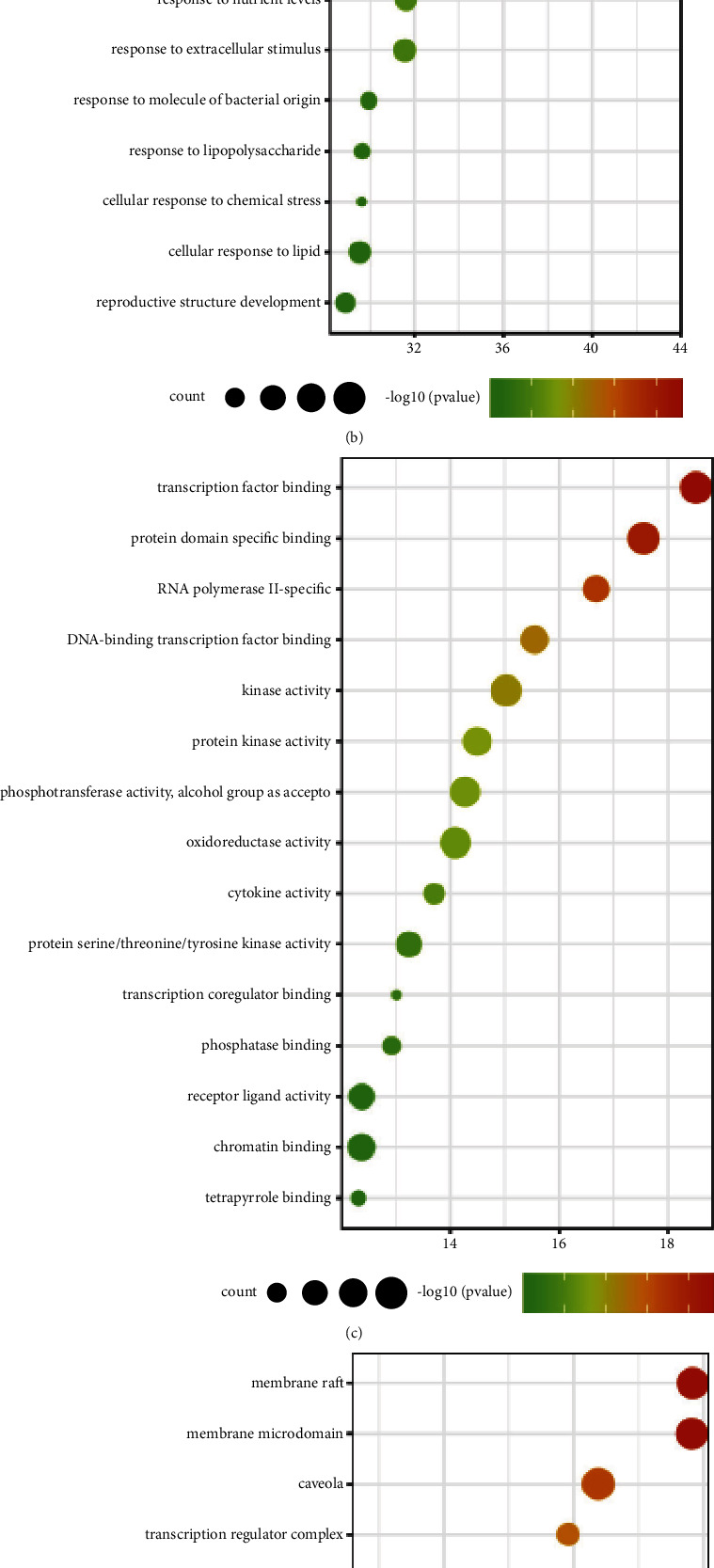
(a) KEGG enrichment analysis: bubble plots of KEGG-enriched immune and endocrine system pathways. Each bubble indicated a KEGG pathway. The size of the bubble was related to the relative ratio of the target to the total target on each pathway, with larger bubbles representing more abundant genes. −log*P* shows the statistical significance of the *P* value. The larger the number was, the more significant the number of the *P* value was. The GO-enriched bubble plot shows the top 15 processes by enrichment, and the abscissa was the value of enrichment, with each bubble representing a GO process. The size of bubbles was positively correlated with the number of targets on each GO, and larger bubbles indicated more abundant genes. −log10 indicated *P* value and color green corresponded to larger *P* values while color red corresponded to smaller *P* values. BP, MF, and CC were shown in Figure (b), (c), and (d), respectively. (a) KEGG pathway. (b) Biological process (BP). (c) Molecular functions (MF). (d) Cell component (CC).

With the help of Metascape database, we selected (*P* value > 0.01, min overlap > 3) and kept more than 5% of the paths with -log (*P* value) > 5, and got 76 KEGG paths. GO process: 177 BP (biological process), 63 CC (cell component), and 93 MF (molecular function). Among KEGG pathways, 16 are related to the immune system and 14 are related to the endocrine system. Immune system pathways mainly involve receptor signaling pathways of T and B cells, Th1, Th2, and Th17 cell differentiation. Endocrine system pathways mainly involve the activation of peroxisome proliferator-activated receptors (PPARs), as well as the synthesis and secretion of hormones. From Figure 7(a), we found that *Scutellariae radix* (Huangqin), *Armeniacae semen amarum* (Kuxingren), and *Peucedani radix* (Qianhu) had more pathways for the immune system and endocrine system intervention, while *Pheretima* (Dilong) only had an effect on the endocrine system. BP was mainly enriched in the regulation of external stimulation and small molecule metabolism, involving the cellular response to inorganic substances, nitrides, lipids, and lipopolysaccharides; MF was mainly enriched in kinase activity, enzyme, and transcription factor binding; CC was mainly enriched in membrane rafts and mitochondrial membranes Table 4.

## 5. Discussion

The properties, flavours, meridian tropism, efficacy, and indications are the standards guiding the use of herbs and it is also the method of applying the theory of TCM to the clinic [16]. In this study, descriptive analysis was used to comprehensively analyze the four properties, five flavours, and meridian tropism of herbs. In general, the frequency of cold herbs is significantly higher than that of warm and mild herbs. The frequency of bitter herbs is significantly higher than sweet and pungent herbs. The theory of TCM indicates that cold and bitter herbs have the function of removing heat from the body, killing and inhibiting pathogenic microorganisms. Warm and pungent herbs have the function of enhancing the body's immune function. Mild and sweet herbs can supplement body nutrition and reduce the toxic and side effects of other flavoured herbs [17–19]. Meridian tropism of herbs refers to the organ targets of herbal components intervention [20]. The study found that the lung meridian, stomach meridian, and spleen meridian were the top three meridian tropisms of prescription herbs. The absorption of human nutrition is mainly in the digestive tract, which is the stomach meridian and spleen meridian in TCM. The location of RRTIs is in the upper and lower respiratory tract, which is also the lung meridian in TCM. The above findings reflect the concept of TCM to remove excess substances in the body and supplement the missing substances to regulate the homeostasis [21]. It conformed to the basic theory of reinforcing healthy qi to eliminate pathogenic factors in TCM.

In clinic, RRTIs can be divided into infectious and noninfectious stages, with complex pathogenesis. In the infectious stage, cough, asthma, fever, and catarrh in the respiratory tract are common. In the noninfectious stage, it is usually associated with pharyngitis, allergic rhinitis, hyperhidrosis, dyspepsia, and other symptoms. TCM physicians will take into account the differences in symptoms during the treatment. Prescriptions vary according to the patient's symptoms. In this study, 100 prescriptions of each physician were collected together for cluster analysis to explore the combined relationship between different herbs. Finally, five effective new prescriptions based on the TCM theory were obtained. Four of these five prescriptions clustered to one physicians, respectively. Of particular interest, New Prescription 3 is a stand-alone set of prescriptions. The herbs in the prescription were commonly used by all four physicians, without being bias towards how one physician. Prescription clustered to physician 1 has the effects of removing stomach heat, promoting gastrointestinal peristalsis, and improving hyperhidrosis and dyspepsia. Prescription clustered to physician 2 has the effects of promoting respiratory sputum excretion and reducing respiratory catarrhal symptoms. The prescription clustered to physician 3 has the effects of improving systemic fever and relieving cough and asthma symptoms. The prescriptions clustered to physician 4 has antiallergic effects and could treat symptoms such as pharyngitis and allergic rhinitis. Prescription 3 is mainly used to treat respiratory tract infections and regulate the body's immune function. These symptoms are consistent with those shown in clinical records for children with RRTIs. Fundamental theory of TCM attaches great importance to the relationship between seasons and disease occurrence. In summary, most illnesses in spring are related to pathogenic wind, and allergic diseases easily occur. It is more appropriate to adopt the prescription of physician 4. Most of the illnesses in autumn and winter are related to pathogenic wind. Children easily suffer from the symptoms of aversion to cold. It is more appropriate to adopt the prescription of physician 3. In addition, treatment based on pattern differentiation is the foundation of the TCM treatment theory. Some studies have pointed out that RRTI has a certain pathophysiological relationship with the frequently occurring combined diseases or symptoms [22]. Therefore, comprehensive treatment of TCM may produce better clinical effect in the process of treating RRTI [23].

Association rule analysis indicated that *Scutellariae radix* (Huangqin), *Armeniacae semen amarum* (Kuxingren), *Peucedani radix* (Qianhu), and *Pheretima* (Dilong) had the closest relationship. They are the core herbs for the treatment of RRTIs. The core herbs were fully consistent with the new prescription 3 found by hierarchical clustering. Therefore, we believed that Prescription 3 may be the core prescription (CP) for the treatment of RRTIs. The mechanism of core prescription for the treatment of RRTIs was studied by KEGG and GO analysis. The four herbs in the core prescription acted synergistically and treat RRTIs in a variety of ways. In China, *Scutellariae radix* (Huangqin) is widely used to treat respiratory tract infections [24]. The active compound (baicalin, wogonin) of *Scutellariae radix* can promote T lymphocyte differentiation and Th1 (IFN-*γ* and IL-12), Th2 (IL-4, IL-5, IL-10, and IL-13), and Th17 (IL-17) cytokine secretion through Th1 and Th2 cell differentiation, Th17 cell differentiation, and T cell receptor signaling pathways in the immune system. Baicalin and wogonin can also exert immunomodulatory and trickling effects by downregulating toll-like receptors through the toll-like receptor signaling pathway, as well as activating the PPAR signaling pathway of the endocrine system [25]. In addition, the active compound of *Scutellariae radix* has antiviral and antibacterial effects. For example, Baicalein can inhibit the replication of COVID-19 virus [26]. The active compound (coumarins, pyranocoumarins) of *Peucedani radix* (Qianhu) can significantly inhibit ovalbumin (OVA)-induced airway inflammation, airway hyperresponsiveness, and Th2 major responses in mice [27, 28]. *Peucedani radix* is commonly used to treat allergic asthma in clinic [29]. A clinical study indicated that *Armeniacae semen amarum* (Kuxingren) was the most common single herb used to treat asthma symptoms in children [30]. Studies had pointed out that *Scutellariae radix* and *Armeniacae semen amarum* could alleviate the symptoms of respiratory tract infection in children and reduce the incidence of RRTIs [30]. Figure 7(a) shows that the endocrine system pathway only enriched by *Pheretima*'*s* targets was PPAR signaling pathway. *Pheretima* could assist *Scutellariae radix* in activating PPAR signaling pathway and exerting immune regulation. In addition, the prescription of TCM containing *Pheretima* could regulate T cell function and attenuate anaphylactic rhinitis-asthma symptoms [31]. According to the theory of TCM, *Scutellariae radix* has the effect of removing lung heat and was the monarch herb in the prescription for the treatment of the most common inflammatory fever symptoms of RRTIs. *Armeniacae semen amarum*, *Peucedani radix*, and *Pheretima* are mainly used to relieve cough, transform phlegm, and relieve asthma. These herbs are used to relieve other syndromes of RRTIs, such as asthma and catarrhal symptoms in the upper respiratory tract. Together with *Peucedani radix*, another herbs also exert the effects of anti-inflammation and immune regulation on the pathway mechanism. It reflects the principle of compatibility (Peiwu) in TCM. This is consistent with the modern medical theory: enhance the efficacy and reduce toxicity [32]. Attention, the clinical prescription should be individualized on the basis of syndrome differentiation and treatment [33]. For example, patients with RRTIs suffering from allergic rhinitis, we can choose a combination of core prescriptions and prescriptions clustered to Physician 4.

Combined with data mining and systems pharmacology, we comprehensively analyzed the prescription patterns of TCM and explained the mechanism of traditional prescription, providing useful new enlightenment for the treatment of RRTIs. In this study, the clinical data and prescriptions of patients were prospectively collected in multiple hospitals in order to ensure the quality of research data and minimize research bias caused by single physician. Through data mining approaches, unique core prescriptions can be discovered from large-scale medical records. In this study, we found that core prescription mainly affects the immune system and endocrine system, which have some significance for further understanding the pathomechanism of RRTIs. However, it has also been suggested that herbal selects for future RRTIs treatment could cover not only the immune system but also the neuroendocrine-digestive system in humans for better clinical outcomes [34]. To some extent, due to the geographical differences of illustrious senior TCM practitioners and the differences in constitutions of children visited, there are still some limitations in this study. In the future, we can further explain the differences in the pathogenesis and treatment mechanism of RRTIs in combination with the genetic genes and living habits of populations in different regions. The safety of the core prescriptions and effectiveness in different stages of RRTIs still need to be assessed by clinical trials, and the mechanism also needs to be further explored and validated by animal experiments.

## 6. Conclusion

Five new prescriptions were found by hierarchical clustering algorithm, and then core herbs were found by the apriori algorithm of association rules. Combining the analysis of the results of the two algorithms, we confirmed the core prescription of *Scutellariae radix* (Huangqin), *Armeniacae semen amarum* (Kuxingren), *Peucedani radix* (Qianhu), and *Pheretima* (Dilong) for the treatment of RRTIs. It mainly promotes the differentiation of Th1, Th2, and Th17 cells and the secretion of inflammatory factors through the toll-like receptor signaling pathway in the immune system, T cell receptor signaling pathway, and PPAR signaling pathway in the endocrine system, to exert immune regulation and anti-inflammatory effects. The research analysis and results indicated that the method used in this study can effectively analyze the mechanism and regularity of TCM prescriptions. In the future, we can further conduct clinical research studies to explore the effectiveness and safety of core prescriptions. At the same time, we can try to find specific monomer compounds in the core prescriptions and study the microscopic mechanism of dual regulation in the immune and endocrine system.

## Figures and Tables

**Table 1:** RRTI diagnostic criteria.

Age (years)	URTI (frequency/years)	LRTI (frequency/years)	
		Recurrent bronchitis	Recurrent pneumonia
0∼2	7	3	2
2∼5	6	2	2
5∼14	5	2	2

*Notes*. (1) The interval between two infections shall be at least 7 days. (2) If the frequency of the upper respiratory tract infections is not enough, the frequency of the upper and lower respiratory tract infections can be added; if the frequency of the lower respiratory tract infections is not enough, they cannot be added up. (3) The frequency shall be confirmed through continuous observation for 1 year. (4) Repeated pneumonia refers to repeated pneumonia ≥2 times within 1 year. Pneumonia must be confirmed by lung signs and imaging. Pneumonia signs and imaging changes should completely disappear during the two pneumonia diagnoses.

**Table 2:** Frequency of herbal characteristics in four physicians' prescriptions.

Herbal characteristics	Types	Physician 1	Physician 2	Physician 3	Physician 4
Four properties	Cold	344	632	604	1001
	Warm	468	282	453	741
	Mild	452	54	173	330
	Cool	27	57	29	45

Five flavours	Sour	182	28	63	76
	Bitter	484	777	864	1081
	Sweet	1075	539	759	891
	Pungent	471	245	484	878
	Salty	109	80	114	88
	Light	39	4	4	1
	Astringent	33	8	9	57

Meridian tropism (top 6 meridians)	Lung	861	1054	1064	1517
	Spleen	585	403	489	552
	Liver	478	228	419	660
	Heart	385	68	215	336
	Kidney	267	48	143	172
	Stomach	740	394	426	793

**Table 3:** Herb characteristics.

Herb	Frequency	Properties	Flavour	Physician
*Scutellariae radix* (Huangqin)	248	Cold	Bitter	1/2/3/4
*Armeniacae semen amarum* (Kuxingren)	234	Warm	Bitter	1/2/3/4
*Glycyrrhizae radix et rhizoma* (Gancao)	215	Mild	Sweet	1/3/4
*Peucedani radix* (Qianhu)	197	Cold	Bitter, pungent	2/3/4
*Magnoliae flos* (Xinyi)	172	Warm	Pungent	1/2/3/4
*Xanthii fructus* (Cang'erzi)	150	Warm	Pungent, bitter	2/3/4
*Ephedrae herba* (Mahuang)	145	Warm	Pungent, bitter	1/2/4
*Pheretima* (Dilong)	142	Cold	Salty	2/3/4
*Trichosanthis fructus* (Gualou)	140	Cold	Sweet, bitter	2/3/4
*Cicadaeperiostracum* (Chantui)	138	Cold	Sweet	2/3/4
*Belamcandae rhizoma* (Shegan)	133	Cold	Bitter	2/3/4
*Crataegi fructus* (Shanzha)	133	Warm	Sour, sweet	1/2/3/4
*Eriobotryae folium* (Pipaye)	132	Cold	Bitter	2/4
*Hordei fructus germinatus* (Maiya)	124	Mild	Sweet	1/2/3
*Massa medicata fermentata* (Shenqu)	102	Warm	Sweet, pungent	1/2/3
*Pinelliae rhizoma* (Banxia)	101	Warm	Pungent	1/2/3/4
*Arctii fructus* (Niubangzi)	97	Cold	Pungent, bitter	1/2/3/4
*Lonicerae japonicae flos* (Jinyinhua)	96	Cold	Sweet	1/2/4
*Asteris radix et rhizoma* (Ziwan)	95	Warm	Pungent, bitter	1/2/3/4
*Platycodonis radix* (Jiegeng)	92	Mild	Bitter, pungent	1/2/3/4
*Descurainiae semen lepidii semen* (Tinglizi)	85	Cold	Pungent, Bitter	2/3/4
*Cynanchi paniculati radix et rhizoma* (Xuchangqing)	85	Warm	Pungent	4
*Cynanchi stauntonii rhizoma et radix* (Baiqian)	84	Warm	Pungent, bitter	1/3/4
*Saposhnikoviae radix* (Fangfeng)	84	Warm	Pungent, sweet	2/3/4
*Fritillariae thunbergii bulbus* (Zhebeimu)	83	Cold	Bitter	2/4
*Ziziphi spinosae semen* (Suanzaoren)	81	Mild	Sweet, sour	1/2/3
*Gastrodiae rhizoma* (Tianma)	80	Mild	Sweet	1
*Lysimachiae herba* (Jinqiancao)	75	Cold	Sweet, salty	1
*Arisaema cum bile* (Dannanxing)	63	Cool	Bitter, pungent	2/4
*Farfarae flos* (Kuandonghua)	60	Warm	Pungent, bitter	2/3/4
*Perillae fructus* (Zisuzi)	59	Warm	Pungent	3/4
*Liquidambaris fructus* (Lulutong)	56	Mild	bitter	4
*Bupleuri radix* (Chaihu)	48	Cold	Pungent, bitter	2/3/4

**Table 4:** KEGG pathways of the immune and endocrine systems.

KEGG	Pathway	Log *P*	Tyle
hsa04062	Chemokine signaling pathway	−14.12	Immune system
hsa04611	Platelet activation	−8.58	Immune system
hsa04613	Neutrophil extracellular trap formation	−9.69	Immune system
hsa04620	Toll-like receptor signaling pathway	−20.49	Immune system
hsa04621	NOD-like receptor signaling pathway	−13.26	Immune system
hsa04622	RIG-I-like receptor signaling pathway	−8.56	Immune system
hsa04623	Cytosolic DNA-sensing pathway	−6.33	Immune system
hsa04625	C-type lectin receptor signaling pathway	−25.17	Immune system
hsa04650	Natural killer cell mediated cytotoxicity	−10.66	Immune system
hsa04657	IL-17 signaling pathway	−27.9	Immune system
hsa04658	Th1 and Th2 cell differentiation	−11.31	Immune system
hsa04659	Th17 cell differentiation	−15.8	Immune system
hsa04660	T cell receptor signaling pathway	−23.58	Immune system
hsa04662	B cell receptor signaling pathway	−16.29	Immune system
hsa04664	Fc epsilon RI signaling pathway	−14.45	Immune system
hsa04670	Leukocyte transendothelial migration	−11.03	Immune system
hsa03320	PPAR signaling pathway	−6.75	Endocrine system
hsa04910	Insulin signaling pathway	−16.76	Endocrine system
hsa04912	GnRH signaling pathway	−11.25	Endocrine system
hsa04914	Progesterone-mediated oocyte maturation	−12.07	Endocrine system
hsa04915	Estrogen signaling pathway	−20.85	Endocrine system
hsa04917	Prolactin signaling pathway	−29.43	Endocrine system
hsa04919	Thyroid hormone signaling pathway	−20.63	Endocrine system
hsa04920	Adipocytokine signaling pathway	−15.91	Endocrine system
hsa04921	Oxytocin signaling pathway	−14.56	Endocrine system
hsa04923	Regulation of lipolysis in adipocytes	−9.46	Endocrine system
hsa04926	Relaxin signaling pathway	−25.99	Endocrine system
hsa04928	Parathyroid hormone synthesis, secretion, and action	−8.11	Endocrine system
hsa04929	GnRH secretion	−10.32	Endocrine system
hsa04935	Growth hormone synthesis, secretion, and action	−15.11	Endocrine system

## Data Availability

All the data used to support the findings of this study are available from the corresponding author upon reasonable request.

## Supplementary Materials

Supplementary Materials Table S1: The difference between related work and previous studies. Table S2: All 45 association rules are shown. Table S3: All 226 disease-herb interaction targets are shown.

## References

[1] Committee C. M. D. A. P. B. A. P. (2016). Clinical diagnosis and treatment pathway of recurrent respiratory tract infection. *Chinese Journal of Practical Pediatrics*.

[2] Wang X., Li X., Jin C. (2021). Association between serum vitamin A levels and recurrent respiratory tract infections in children. *Front Pediatr*.

[3] Patria M. F., Esposito S. (2013). Recurrent lower respiratory tract infections in children: a practical approach to diagnosis. *Paediatric Respiratory Reviews*.

[4] Verwey C., Nunes M. C., Dangor Z., Madhi S. A. (2020). Pulmonary function sequelae after respiratory syncytial virus lower respiratory tract infection in children: a systematic review. *Pediatric Pulmonology*.

[5] de Vries E., Soc C. E. (2011). Patient-centred screening for primary immunodeficiency, a multi-stage diagnostic protocol designed for non-immunologists: 2011 update. *Clinical and Experimental Immunology*.

[6] Diez-Domingo J., Perez-Yarza E. G., Melero J. A. (2014). Social, economic, and health impact of the respiratory syncytial virus: a systematic search. *BMC Infectious Diseases*.

[7] Tian Y., Wang L., Wang Z. (2021). Efficacy and safety of Tuina for treatment of pediatric recurrent respiratory tract infections A protocol for systematic review and meta-analysis. *Medicine (Baltimore)*.

[8] Song T., Hou X., Yu X. (2016). Adjuvant treatment with yupingfeng formula for recurrent respiratory tract infections in children: a meta-analysis of randomized controlled trials. *Phytotherapy Research*.

[9] Changquan Y. (2012). Clinical study on TCM treatment optimization scheme for recurrent respiratory tract infection in children. *Chinese Journal of Traditional Chinese Medicine*.

[10] Bu D. C., Xia Y., Zhang J. (2021). FangNet: mining herb hidden knowledge from TCM clinical effective formulas using structure network algorithm. *Computational and Structural Biotechnology Journal*.

[11] Chen H. Y., He Y. (2022). Machine learning approaches in traditional Chinese medicine: a systematic review. *The American Journal of Chinese Medicine*.

[12] Zhang R. Z., Yu Sj, Bai H., Ning K. (2017). TCM-Mesh: the database and analytical system for network pharmacology analysis for TCM preparations. *Scientific Reports*.

[13] Li X. (2012). Summary of professor Guo zhenwu’s experience in treating recurrent respiratory tract infection in children. *Journal of Liaoning University of Traditional Chinese Medicine*.

[14] Changyong L. (2021). Multi-dimensional analysis of professor Li suqing’s regularity in treating children with recurrent respiratory tract infection. *Chinese Journal of Traditional Chinese Medicine Information*.

[15] Qiu J. (2007). A culture in the balance. *Nature*.

[16] Fu J. L., Pang J., Zhao X., Han J. (2015). The quantitative ideas and methods in assessment of four properties of Chinese medicinal herbs. *Cell Biochemistry and Biophysics*.

[17] Fu X. J., Mervin L. H., Li X. (2017). Toward understanding the cold, hot, and neutral nature of Chinese medicines using in silico mode-of-action analysis. *Journal of Chemical Information and Modeling*.

[18] Park S. M., Baek S. J., Ban H. J., Jin H. J., Cha S. (2022). Systematic analysis of the molecular mechanisms of cold and hot properties of herbal medicines. *Plants*.

[19] Liu J., Feng W. W., Peng C. (2020). A song of ice and fire: cold and hot properties of traditional Chinese medicines. *Frontiers in Pharmacology*.

[20] Juan W. (2016). Progress and ideas of modern research on meridian introduction theory of Chinese medicine. *Chinese Journal of Traditional Chinese Medicine*.

[21] Li P. P. (2012). Toward an integrative framework of indigenous research: the geocentric implications of Yin-Yang Balance. *Asia Pacific Journal of Management*.

[22] Schaad U. B., Esposito S., Razi C. H. (2015). Diagnosis and management of recurrent respiratory tract infections in children: a practical guide. *Archives of Pediatric Infectious Diseases*.

[23] Lining W. (2008). Guide to TCM diagnosis and treatment of recurrent respiratory tract infection in children. *Journal of Traditional Chinese Medicine*.

[24] Zhao Q., Chen X. Y., Martin C. (2016). Scutellaria baicalensis, the golden herb from the garden of Chinese medicinal plants. *Science Bulletin*.

[25] Liao H. F., Ye J., Gao L., Liu Y. (2021). The main bioactive compounds of Scutellaria baicalensis Georgi. for alleviation of inflammatory cytokines: a comprehensive review. *Biomedicine & Pharmacotherapy*.

[26] Pei T. L., Yan M., Huang Y., Wei Y., Martin C., Zhao Q. (2022). Specific flavonoids and their biosynthetic pathway in scutellaria baicalensis. *Frontiers of Plant Science*.

[27] Xiong Y. Y., Wu F. H., Wang J. S., Li J., Kong L. Y. (2012). Attenuation of airway hyperreactivity and T helper cell type 2 responses by coumarins from Peucedanum praeruptorum Dunn in a murine model of allergic airway inflammation. *Journal of Ethnopharmacology*.

[28] Chu S. S., Chen L., Xie H. (2020). Comparative analysis and chemical profiling of different forms of Peucedani Radix. *Journal of Pharmaceutical and Biomedical Analysis*.

[29] Huang T. P., Liu P. H., Lien A. S. Y., Yang S. L., Chang H. H., Yen H. R. (2013). Characteristics of traditional Chinese medicine use in children with asthma: a nationwide population-based study. *Allergy*.

[30] Yang F. S., Gao F., Tan T. H. (2020). Prescription and medication regularity of traditional Chinese medicine for treating child pneumonia based on data mining. *Zhongguo Zhong yao za zhi = Zhongguo zhongyao zazhi = China journal of Chinese materia medica*.

[31] Zhou K. L., Liu L., Shi S. F. (2014). Qu Feng Xuan Bi Formula attenuates anaphylactic rhinitis-asthma symptoms via reducing EOS count and regulating T cell function in rat ARA models. *Journal of Ethnopharmacology*.

[32] Zhang J. H., Zhu Y., Fan Xh, Zhang Bl (2015). Efficacy-oriented compatibility for component-based Chinese medicine. *Acta Pharmacologica Sinica*.

[33] Jiang M., Lu C., Zhang C. (2012). Syndrome differentiation in modern research of traditional Chinese medicine. *Journal of Ethnopharmacology*.

[34] Luo C. Y., Yu H., Yang T. (2020). Data mining and systematic pharmacology to reveal the mechanisms of traditional Chinese medicine in recurrent respiratory tract infections’ treatment. *Evidence-based Complementary and Alternative Medicine*.

